# Mechanisms Underlying the Bioindicator Notion: Spatial Association between Individual Sexual Performance and Community Diversity

**DOI:** 10.1371/journal.pone.0022724

**Published:** 2011-07-26

**Authors:** Paola Laiolo, María J. Bañuelos, Beatriz Blanco-Fontao, Mónica García, Gloria Gutiérrez

**Affiliations:** Research Unit of Biodiversity (CSIC, UO, PA), Oviedo University, Oviedo, Spain; University of Western Ontario, Canada

## Abstract

The bioindicator notion is an appealing concept that has received more support in applied than in basic ecology, mostly due to the difficulty in deriving general ecological rules applicable to all target organisms. However, recognizing the mechanisms that determine the association between a particular species and the well-being of many other species is important for understanding the functioning of ecosystems and the relationship among different biological levels. We examined here the processes at the individual level that cause an association between species performance and biodiversity value, by analyzing attributes that can be studied in a variety of animals with sexual reproduction, namely breeding site selection and condition-dependent sexual signals. Our study model was the Capercaillie, an indicator of forest functioning and diversity, and the associated bird community, used here as a surrogate of broader forest biodiversity. At a regional scale Capercaillie occurrence was not associated with the most diverse forest patches, but at the scale of male spring territories the sexual display grounds (arenas) were located in the oldest and less disturbed forest portions, which also hosted the richest local bird communities. Social mechanisms and conspecific cueing likely concurred with habitat-driven processes in determining the long-term persistence of traditional display grounds, which were appealing to many other species because of their structural composition. Characteristics of male vocal display that honestly advertize male quality (low frequencies and rapid song rates) were significantly correlated with high diversity values, resulting in a spatial association between individual and community performances. Costly or risky activities such as reproductive or social behaviors, which more than other attributes match gradients in habitat quality, are therefore contributing to functionally connect individuals with ecosystem health.

## Introduction

Habitat-related processes play a key role in evolutionary and ecological dynamics. Major factors contributing to habitat quality have a direct effect on individual fitness and drive evolution, and the interplay between habitat and populations, communities or food webs constitutes the base of the ecological theory [1]. Recent integrative studies have demonstrated cascading effects between evolutionary aspects relevant at the individual level and community properties [2], [3]. As an example, the effects of any stressor – abiotic or biotic, natural or anthropogenic – on individuals within populations can cascade through direct and indirect interactions to any other population that shares the same ecosystem [4]. Mechanistic approaches to community ecology tend to infer causality and a direct feedback between levels of the biological hierarchy. A more intuitive case in which community and individual traits may be linked, however, is represented by spurious (noncausal) covariation, occurring when both community and individual performances depend on the same key features of the habitat, on its productivity or stability [5]. This individual-community coupling reflecting habitat quality or health stands at the base of the concept of surrogate species (including indicator, umbrella, keystone and flagship species [6], [7]). The latter are often spatio-temporally associated with community complexity, and only in some cases causatively promote high diversity (through resource facilitation, trophic cascades, etc.) [8]–[10].

Individual features related with habitat and patch selection, or with breeding performance, are good candidate mechanisms determining species-biodiversity association, because they are intrinsically linked with species distribution and population dynamics [11], [12]. Although habitat cues tend to be species-specific, habitat selection and spatial behavior may link the occurrence of one species with that of other with similar requirements and determine a species to be spatially associated with diverse communities, especially when several species perform better in habitats that are highly productive or complex [13]. Diffuse human disturbance, for instance, may concentrate intolerant species together at a limited number of undisturbed sites, most of them presenting high conservation value [8]. Sexual behaviors may also drive concordance among community members, given that their effects on breeding success may covary among species within the same local assemblage [14].

The potential role of individual traits in mechanistically linking a species and its surrounding community has never been explicitly addressed in spite of being important in both basic and applied ecology, for understanding the functioning of ecosystems and for testing the effectiveness of the ‘surrogate’ label given to species [5], [15]. In this study we analyzed the relationship between the variation in condition-dependent and social behaviors at the individual level and the diversity patterns of the associated community. The question originates from the hypothesis that habitats characterized by stability in ecological conditions or by maturity (i.e. close to an equilibrium point), may positively feedback to the performance of species inhabiting them, which at the same time would form complex and diverse assemblages [16]. We centered this study on the sexual display of a bird species, the Capercaillie *Tetrao urogallus*, characterized by a polygynous mating system in which males strongly compete for good breeding areas and females. Intrasexual competition is the driver of the large sexual size dimorphism in Capercaillie and exaggerated display, which honestly indicate male quality [17]. Under polygyny conditions, males are expected to vary greatly in their quality and their capability to monopolize the best breeding areas, resulting in a co-variation between individual and habitat quality and consequent disparity in male breeding success [18].

A variety of studies have classified the Capercaillie as an indicator, umbrella or keystone species of forest bird diversity, ecosystem health, functioning or ‘naturalness’ [19]–[22]. This grouse inhabits boreal or montane mature forest, one of the most diverse Palearctic habitats that has been severely affected by human activities for centuries [23]. In this study, we used bird diversity as a surrogate of forest community diversity, since birds are known for responding to broad ecosystem characteristics in a manner similar to other taxa [24], and at the small scale diversity is often higher in undisturbed, mature forests than in disturbed ones [15], [25]. We first tested whether the regional distribution of the Capercaillie was associated with bird diversity at the scale of forest patch. Then, we tested whether species selection of specific areas within their territories (namely the display ground, hereafter ‘arena’) was spatially-associated with bird diversity, taking into account that displaying males show breeding site fidelity within and among generations, and are vulnerable to anthropogenic disturbance [26], [27]. Finally, we analyzed the association between bird diversity and Capercaillie song acoustic characteristics related to male quality, not to propose a shortcut to measure habitat complexity via the quantitative study of such characters, but rather to investigate the functional link between surrogate species characteristics and the community context they reveal.

## Materials and Methods

### Ethics Statement

The Capercaillie is an extremely imperiled species in Spain, and access to male arenas is strictly controlled by regional administrations. For this study, the authors obtained permissions from the Asturian Regional agency to visit a maximum of fourteen arenas located in 10 known territories to record male vocalizations (Permission 2010/001409 of the Asturian Regional Government, including access to Natural Park of *Fuentes del Narcea, Degaña e Ibias*, and the *Natural Reserve of Muniellos*). To minimize disturbance when recording, we reached male arenas 1–1.5 hr before dawn (when display usually starts) and left it after 30 min of behavioral observations and 10 min of bird survey (see below). Avifauna surveys (point-counts) were not subject to restrictions in our study area.

### Study area and species

**Figure 1:**
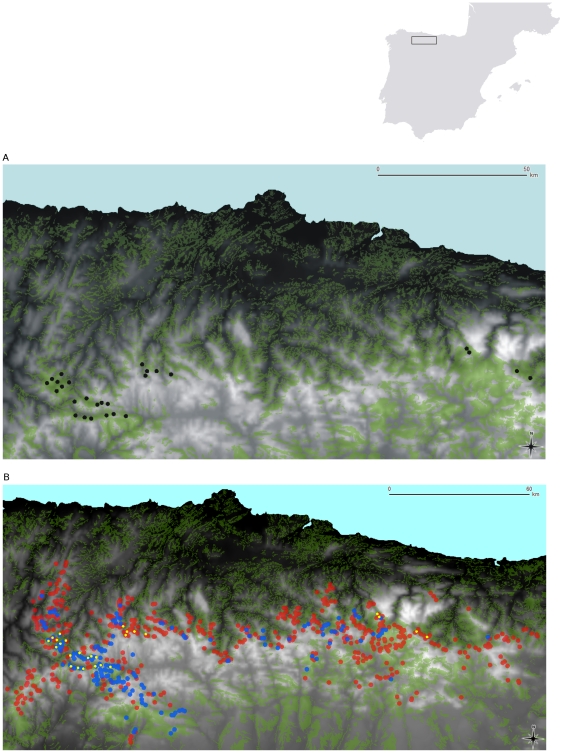
Study area and Capercaillie distribution in Cantabrian Mountains. (A) Location of the 27 study plots (black dots). Green areas represent wooded habitats, the grey colour scale denotes the altitudinal gradient (lighter at higher altitudes). (B) Capercaillie home ranges in 2005–2006 (blue areas), abandoned plots (red areas) and study plots (yellow dots).

We performed this study in several locations across the Cantabrian Mountains (NW Spain), in mature woodlands that occurred within the Capercaillie range in the 1970s but only patchily inhabited by this grouse at present [26] (Figure 1). The Cantabrian Capercaillie has been experiencing a sharp decline over the last three decades; details on the conservation status and evolutionary and ecological differentiation of this population can be found in Blanco-Fontao *et al.* [22] and Storch *et al.* [28]. Cantabrian montane forests have a broadleaf canopy dominated by beech (*Fagus sylvatica)*, mountain birch (*Betula pubescens)* and oaks (*Quercus petraea* and *Q. pyrenaica)*; the understory includes hazelnut (*Corylus avellana)*, holly (*Ilex aquifolium)*, rowan (*Sorbus aucuparia)*, and medium - small size shrubs of the genus *Vaccinium*, *Erica*, *Rubus*, and *Cytisus*.

Capercaillie spring home ranges (20–75 ha in our study area) include the breeding, feeding and resting spots of cocks, and those of their female mates, often closely located [29]. Within this area, the ‘arena’ is found, a small territory where cocks consistently display for hens (ca 0.30 ha; [29]). Arenas in the Cantabrian population are generally attended by one displaying male alone that aggressively defends its territory from intruders, and territorial turnover among neighbors is uncommon [29]. Although true lekking is not common, for simplicity we use the term ‘arena’ throughout the paper to define the location where males perform their display.

Capercaillie display areas in Cantabrian Mountains have been surveyed since the 1980s by the regional environmental agencies. In spring 2010, we performed dawn surveys in several of the known territories, in order to localize displaying males, update information on occupancy and male numbers, and record male songs during displays (details below).

### Parameters associated with Capercaillie presence and bird diversity

In April-June 2010, we surveyed twenty-seven 50-ha forest plots that included both currently occupied (10 plots, hereafter named “Capercaillie plots”) and abandoned (17 plots, hereafter “abandoned plots”) Capercaillie home ranges. Within each plot, we placed 6–12 census stations of 50 m radius where we measured bird abundance and richness by means of 10-min point counts [30]. We positioned points within plots at least 100 m apart along randomly located transects; for some wide-ranging species, such as woodpeckers, cuckoos and jays, we were careful not to record the same individuals on neighboring points by tracking individual short-range movements [25]. In Capercaillie plots, we placed one census station where males performed their display (see below). In all Capercaillie plots we found fresh signs of the species presence (droppings, feathers) or we saw birds during the 2010 field work season; plots were considered as abandoned where no Capercaillie sign was found.

**Figure 2:**
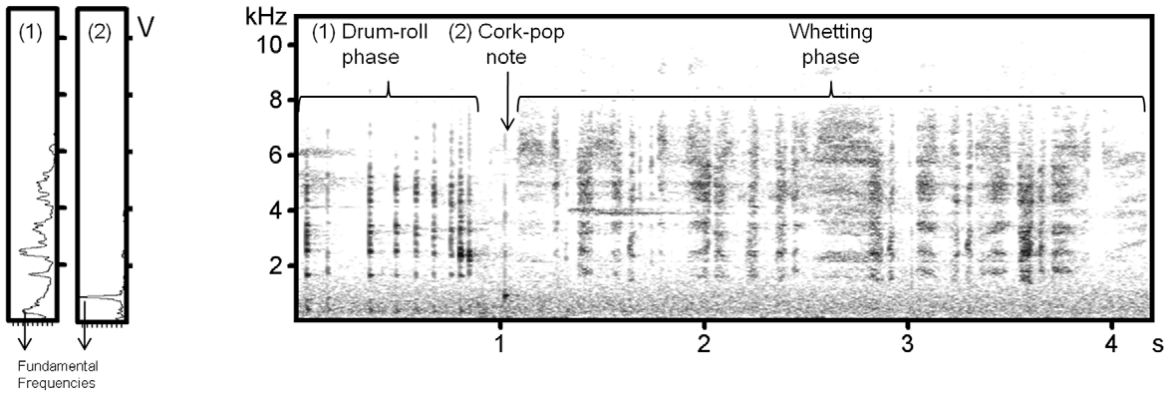
Spectrogram and mean spectra of Capercaillie song. Example of a spectrogram of a Capercaillie song (right) and mean spectra of the first click of the drum-roll phase (1) and of the cork-pop note (2) (left). Arrows indicate the spectral parameters taken into account for this study (fundamental frequency of the drum-roll and of the cork-pop). In the time domain, we measured the duration of the drum-roll and whetting phase.

After each point count was completed, habitat data were collected in circular plots of 20 m radius centred on each avian census station. To describe forest structure and composition, we measured ten parameters that are known to affect forest bird diversity and Capercaillie occurrence, and indicate forest age (tree diameter, number of dead trees) and structural or taxonomic complexity (shrub cover, shrub and tree species richness, etc.) (Figure 2) [31], [32]. A total of 236 point count stations were distributed in 27 forest plots in the altitudinal range of 830–1600 m a.s.l. A list of the bird species detected is provided in Table S1. As expected, forest specialists [33] dominated the study community, given that census plots were located in continuous forests that have not been managed at least since the 1980s.

Bird diversity per census station was calculated with the Shannon-Weiner diversity index, thus taking into account both species occurrence and abundance (H' = -Σp*_i_*×ln p*_i_*, where p*_i_* is the relative frequency of observations of species *i*). Before performing statistical tests, diversity values were transformed by means of a Box–Cox transformation (y = (x^λ^−1)/λ, λ = 4.88) to attain a normal distribution.

Bird diversity per forest plot was estimated as the total number of bird species detected in the census stations of each plot. This estimate did not vary with the number of census stations per plot (from 6 to12) or with its square value (generalized linear models with a Poisson distribution of errors, GLM, all *Z*<0.32, *P*>0.71, *n* = 27 plots). Species richness per forest plot and the average Shannon-Weiner diversity index per station within that plot were not significantly correlated (GLM, *Z* = 1.4, *P* = 0.14, *n* = 27 plots) and were thus used as independent measures of community diversity.

### Capercaillie sexual display

We reached male arenas 1–1.5 hr before dawn. Once we detected a male in the initial phase of the song (the tapping phase, an incomplete song given from a perch that after several minutes develops into the complete song), we cautiously approached singing males, up to a distance of 15 m ca; one observer remained there recording until they spontaneously stopped singing or left voluntarily, in order to avoid any disturbance.

Arenas were checked from late April to early June, but recordings were obtained between the 8^th^ and the 25^th^ of May, when male sexual activity peaked. In this period, each arena was visited on average 2.3±0.3 SE times (1–4 visits per arena) until recordings were obtained. For 10 males from 8 separate forest plots (territories) we obtained good recordings, i.e. with a good signal-to-noise ratio. Each arena was visited by two of us, one recorded the target male and the other searched for other potential display grounds in the area, to estimate the number of displaying males and the position of all the active arenas in a plot. Birds were not marked, but we can reasonably exclude that the same male was recorded in different arenas, because arenas were located in mountainous areas far from each other, male turnover is reduced in the Cantabrian area [29], two or more observers' group contemporary checked and recorded in different arenas, and only data from 1 recording-day per male were used, to avoid potential problems of recording the same male in different sessions [34]. Birds were recorded with a Marantz PMD 660 recorder, and a Sennheiser ME67 microphone (frequency response 50–20 000 Hz). In the exact location at which the male was displaying (either recorded or not), we centered a biodiversity census station.

Sound analyses were carried out with Avisoft SASLab Pro Software by Raimund Specht (Berlin), performing a Fast Fourier Transform (sampling frequency 22050 Hz, FFT length 1024, time resolution 3 msec, frequency resolution 22 Hz, Window Function: Bartlett). Acoustic characteristics were measured in the time domain on envelopes and in frequency domain on fast Fourier transform mean spectra. We measured the duration of the drum-roll phase, the duration of the whetting phase, the fundamental frequency of the first click of the drum-roll phase and the fundamental frequency of the cork-pop note (Figure 2). Measurements were taken on 100 songs, 10 per individual. Acoustic variables did vary among individuals (one-way ANOVAs: 3.1<*F*<8.2, all *P*<0.005) but were repetitive within individuals (intra-individual correlation coefficients ranged from 0.20 to 0.62, all *P*<0.005 [35]). We can thus consider that study males differed sufficiently in their songs for selection to act [36]. Mean values per individual of each acoustic variable were used in the analysis.

As a descriptor of Capercaillie display, we also took into account the number of complete songs given per minute during the high intensity acoustic display, i.e. since males started giving the complete song from a perch until they ventured on to the ground. In this perch phase, song rate is highly constant through time if the bird is not disturbed [29]. On the ground, individuals add visual signals and non-vocal sounds (flutter-jumps, beard and shoulder-spot displaying, etc.) and their vocal activity becomes irregular; for this reason, we did not consider incomplete vocalizations given from the ground for estimating song rate and only considered the perch song.

All in all, Capercaillie song was described by a set of acoustic variables that are commonly used to represent the songs of this species or of other bird groups and that often correlate with male quality, health condition, and competition levels [34], [37], [38]. As an example, the fundamental and minimum frequencies can be negatively related to body size, a sexually selected trait in Capercaillies [39], [40], whereas song rate or duration may reflect male condition or endocrine status [41]. Displaying Capercaillie males from neighboring grounds are known to stimulate each other by increasing song rates [29], [42].

### Community diversity and Capercaillie occurrence at the regional scale: comparison between occupied and abandoned forest plots

By using the whole data set of 236 point counts, we tested whether Capercaillie occurrence at the forest plot level was associated with the diversity of birds in census stations. We used generalized linear mixed models (GLMMs) with a binomial distribution of errors (0 = stations in abandoned plots, 1 =  stations in Capercaillie plots) and a logit link function. The identity of the 27 sample plots was entered as a random variable, and geographic coordinates were entered as covariates to further control for spatial autocorrelation and nonindependence of stations located within the same plot. With the same dataset, we analyzed the environmental determinants of Capercaillie occurrence and bird diversity per station, by means of GLMMs with a binomial and Gaussian distribution of errors, respectively. As predictors, we entered altitude and the habitat variables listed in Table 1. Geographic coordinates were entered as covariates, and plot identity as a random factor.

**Table 1:** List of habitat variables measured per census station.

Variable	Measurement	Species
Percentage of tall shrub	Percentage of shrubs >1.7 m high in a radius of 20 m.	*Ilex aquifolium*, *Taxus baccata*, *Corylus avellana*, *Sorbus aria*, *Sorbus aucuparia*,*Sambucus nigra*, etc.
Percentage of medium size shrub	Percentage of shrubs 0.5–1.7 m high in a radius of 20 m.	*Erica arborea*, *Erica australis*, *Cytisus scoparius*, *Daphne laureola*, etc.
Percentage of low shrub	Percentage of shrub <0.5 m high in a radius of 20 m.	*Vaccinium myrtillus*, *Rubus* spp., etc.
Shrub species richness	Number of species of high, medium and low shrub	See above
Canopy diversity	Canopy trees in a radius of 20 m were counted and identified. Proportions were used to calculate Shannon Diversity index of canopy trees (H' = -Σp*_i_*×ln p*_i_*, where p*_i_* is the relative frequency of observations of species *i*).	*Quercus pyrenaica*, *Quercus petraea*, *Fagus sylvatica*, *Acer platanoides*, *A. pseudoplatanus*, *Fraxinus excelsior*, *Alnus glutinosa*, *Salix* spp.
Tree diameter at breast height (DBH)	The diameters of the closest four canopy trees around the observer, one within each quartile (north, south, east and west) were measured with a tape. The four measurements were summarized as mean values per station.	
Canopy openness	We paced in meters the distance between the four closest trees (see DBH). The four measurements were summarized as mean values per station	
Variability in tree diameter	Coefficient of variation (S.D./mean ×100) of the four DBH measurements per station	
Variability in tree openness	Coefficient of variation of the four distance measurements per station	
Number of dead trees	Number of dead trees (standing and fallen) in a radius of 20 m.	

Generalized linear models (GLMs) were run to test for the association between overall bird species richness per plot and Capercaillie occupancy. A binomial distribution of errors (0 = abandoned plot, 1 = Capercaillie plot) was used, and geographic coordinates were entered as covariates in the analysis.

### Community diversity and Capercaillie selection of display grounds: comparison between arenas and other areas within spring home ranges

Previous studies from our study area and other regions found that adult males tend to restrict their spring movements within 1 km of the arena; the latter area is attended with great fidelity every year and across generations [29], [43], [44]. In keeping with the literature, we analyzed bird diversity variation within male territories up to 1 km from the arena, limiting the analysis to the area potentially used by a displaying male (arena, plus feeding and resting areas).

In Capercaillie plots, we calculated the closeness of each station to the arena, and analyzed how bird diversity varied with such distance. We tested for differences in bird diversity between stations located in arenas and in areas not used for displaying. We also analyzed the habitat determinants of bird diversity, and habitat differences between arenas and other portions of Capercaillie home range. We ran GLMMs with a binomial (1 = arena, 0 = no-display ground) and Gaussian (bird diversity) distribution of errors. Plot identity was entered as a random factor, geographic coordinates, altitude and habitat variables (Table 2) as predictors.

**Table 2:** Habitat determinants of Capercaillie occurrence and bird species diversity as highlighted by GLMMs.

Determinants of Capercaillie occurrence			
	Estimate	SE	Z-value
Intercept	−1.48	0.68	−2.1*
Diversity of arboreal species	2.00	0.8	2.5*
Percent cover of high shrub	−0.05	0.02	−2.8**
Latitude	−0.003	0.0005	−3.9***
Longitude	0.27	0.14	2.00**
AIC = 181			

Determinants of bird diversity per census station			
	Estimate	SE	t-value
Intercept	0.21	0.004	5.5***
Number of dead trees	0.045	0.02	2.6**
(Number of dead trees)^2^	−0.005	0.002	−2.0*
Percent cover of low shrub	−0.0013	0.0005	−2.4*
Shrub species richness	0.03	0.009	3.4**
Average tree diameter	0.03	0.0001	3.2**
Latitude	−0.23	0.0001	−3.2**
Longitude	0.50	0.001	−3.3**
AIC = −95			

Other models were separated by >2 AIC points from those shown here.

*P<0.05.

**P<0.01.

***P<0.001.

### Community diversity and Capercaillie sexual performance

The relationship between song parameters and bird diversity in the arena census station and in the forest plot was analyzed by means of GLMs, with a Gaussian distribution of errors since acoustic variables were normally distributed.

In statistical tests, sample size corresponded to 236 census stations (for Shannon Wiener diversity index) and 27 forest plots (overall bird species richness) when comparing Capercaillie and abandoned plots, to 70 stations when comparing arenas *versus* no-display grounds in Capercaillie plots, and to 10 individuals when testing for the association between acoustic parameters and diversity. When analyzing bird diversity/Capercaillie occurrence *vs*. habitat characteristics, model selection was based on the Akaike's Information Criterion (AIC). All statistical analyses were performed with R [45].

## Results

### Community diversity and Capercaillie occurrence at the regional level

**Figure 3:**
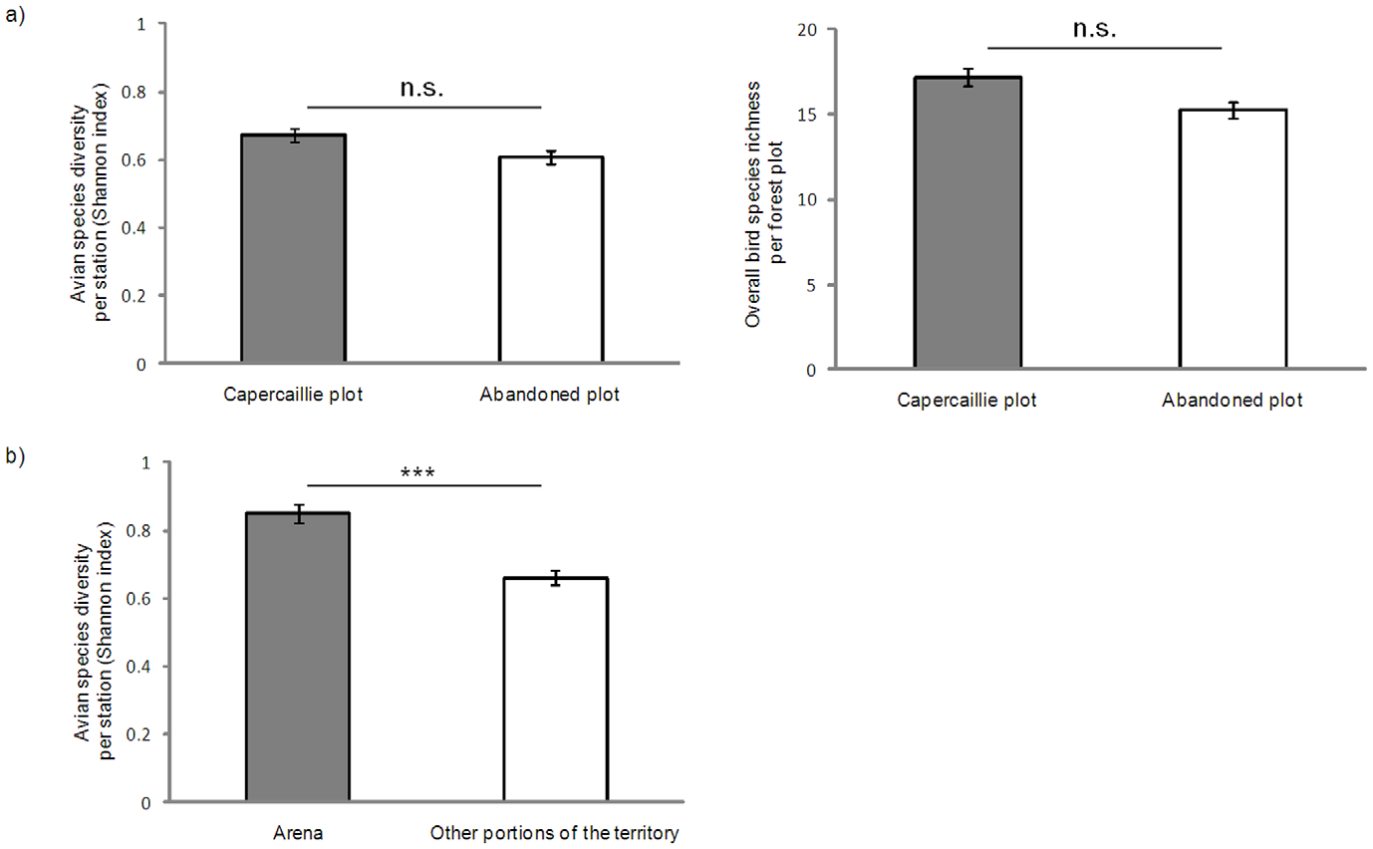
Bird diversity in Capercaillie plots, abandoned plots and arenas. Differences in bird species diversity per census station and overall species richness between Capercaillie plots and abandoned plots (a), and between Capercaillie arenas and other portions of its territory (b). Mean and SE are shown. *** P<0.001.

No significant association was found between Capercaillie occurrence and bird diversity as measured by Shannon-Wiener index per station (GLMM: *Z* = 0.05, *P* = 0.95, *n* = 236 stations) or by overall bird species richness per forest plot (GLM: *Z* = 1.1, *P* = 0.25, *n* = 27 plots) (Figure 3a). Capercaillie occurrence and bird diversity were determined by different habitat predictors: Capercaillie plots had significantly greater canopy species richness and a smaller proportion of high shrubs with respect to abandoned plots, whereas bird diversity peaked in stations characterized by high shrub species richness, large trees, abundance of dead trees and scarce cover of low shrubs (Table 2)

### Community diversity and Capercaillie selection of arenas

Bird diversity peaked in plots located in the arena (GLMM: *Z* = 3.2, *P*<0.001, *n* = 70 stations) (Figure 3b) and decreased at increasing distances, although not linearly (GLMM: distance: *Z* = −2.46, *P* = 0.016; distance ^2^: *Z* = 2.1, *P* = 0.04; *n* = 70). Capercaillie arenas were usually located in the oldest forest stand of the plot. These stations were characterized by the largest living trees, where bird diversity increased as well (Table 3).

**Table 3:** Habitat determinants of Capercaillie choice of arenas, and of bird diversity in Capercaillie territories, as highlighted by GLMMs.

Factors that determine arena choice in the Capercaillie			
Model 1	Estimate	SE	Z value
Intercept	−23.3	9.4	0.13
Tree diameter	0.45	0.21	2.1*
(Tree diameter)^2^	−0.004	0.002	−1.9·
Cover of medium size shrub	0.15	0.09	1.8·
Cover of medium size shrub×tree diameter	−0.004	0.003	−1.7·
Altitude	0.008	0.005	1.7·
AIC = 64.1			

Model 2	Estimate	SE	Z value
Intercept	−10.8	4.58	−2.3*
Tree diameter	0.4	0.20	2.0*
(Tree diameter)^2^	−0.003	0.002	−1.71 ·
Cover of medium size shrub	0.15	0.085	1.80·
Cover of medium size shrub × tree diameter	−0.004	0.003	−1.69·
AIC = 65.5			

Determinants of bird diversity in Capercaillie territories			
	Estimate	SE	t value
Intercept	0.26	0.05	5.6***
Tree diameter	0.004	0.001	3.2**

The effects of latitude and longitude (or square terms) were not significant, and therefore were excluded from the analysis. Only models separated by <2 AIC points from the best models are shown.

*P<0.05,

***P<0.001, P<0.1.

AIC =  −36.93.

### Community diversity and Capercaillie vocal display

Song rate was significantly associated with the number of displaying males (i.e., the number of active arenas in a plot; GLM: β± SE  = 4.6±0.4, *t*_10_ = 2.5, *P* = 0.039) and with geographic coordinates (latitude; β± SE  = 0.0007±0.000, *t*_10_ = 2.5, *P* = 0.038). The other acoustic parameters were no significantly affected by the above variables, and the period of recording (over 18 days of displays) had no effects either. In the light of the above results, latitude and male number were entered in song rate models to control for their potential confounding effects.

**Figure 4:**
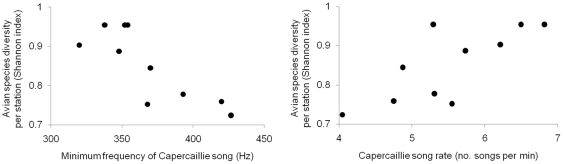
Relationships between Capercaillie song attributes and bird diversity. Bird species diversity decreases where the minimum frequency of Capercaillie song increases, and where Capercaillie sings at a faster rate.

Two acoustic variables of Capercaillie songs were significantly associated with bird diversity in display grounds: song rate (GLM controlling for the number of displaying males and latitude: β± SE = 7±2; *t*_10_ = 4.2, *P* = 0.006) and the fundamental frequency of the drum-roll phase (GLM: β± SE = −311±76; *t*_10_ = 4.1, *P* = 0.0036), i.e. the lowest frequency of the whole Capercaillie vocalization (Figures 2, 4). The latter spectral parameter was also associated with overall species richness in Capercaillie plots (GLM: β± SE = −22±9, *t*_10_ = 2.7; *P* = 0.020), suggesting that males singing the lower pitched songs settled in stations and plots where bird diversity was highest. Male singing at low frequencies displayed in arenas where the cover of tall shrubs was high (GLM *t*_10_ = 5.3; *P* = 0.0007).

In spite of the small sample, there is no apparent inflation of the overall Type I error rate (i.e., rejecting the null hypothesis when it is true) since significance is maintained after performing Bonferroni sequential correction (setting P-threshold to 0.013 and 0.02, i.e. four tests). Notably, the probability of increasing type-II error (false-negative) is the most severe problem of small samples, implying that the relationships we found are robust but the overall number of significant trends might have been underestimated.

## Discussion

We found that the richness of the bird community and aspects of Capercaillie behavior such as breeding site selection and song properties were positively associated. This coupling was dictated by habitat-mediated processes acting at the individual and community levels: Capercaillie arenas were located in the oldest and less disturbed stands, features that make these areas appealing to a variety of forest bird species as well. The most diverse communities were found in old forest spots and in arenas where cocks sang the lowest pitched songs and displayed with the greatest intensity, both aspects being indices of sexual performance quality.

### Patters at the regional scale

Occurrence cannot be considered the parameter that confers the status of umbrella species to the Capercaillie in the Cantabrian range, at least when considering the association with the assemblage of forest birds at the regional scale. The large decline of the species in the last decades has not been accompanied by major habitat changes, and species regression and present distribution may be governed by drivers other than the habitat alone, such as historical or demographic factors [27]. Indeed, a few habitat descriptors clearly explain the current occupancy of Capercaillie display areas, in contrast to bird species diversity, which responds to a variety of microstructural and floristic features, in general revealing forest age. As in many other European forests, at the small scale of individual territories the richest bird communities are found in the oldest stands, rich in dead wood, with a complex shrub layer and large trees offering plenty of nesting and feeding resources for cavity nesting and insectivorous birds [25], [32], [46]. In temperate latitudes, stability and conditions closer to a ‘natural’ state (*sensu* Anderson [47]) confer mature forests a key role for the conservation of biodiversity and the ecological dynamics that the latter maintain [48].

### Patterns at the scale of male territories

Arenas are high diversity spots within the forest, and diversity decreases at increasing distance from this small area. The habitat feature that arenas share with species-rich areas is large tree size (diameter), a finding that emphasizes the importance of forest age for Capercaillie sexual performance and for the rest of the forest bird community [33], [49]. Considering the small size of arenas and the wide habitat requirements of this large grouse during breeding, however, it seems improbable that arena selection is dictated solely by habitat processes [50]. A complementary explanation is provided by social mechanisms, which may assist the Capercaillie in arena selection. It is known that cocks defend their grounds with few shifts from year to year, and when the dominant male dies its place tends to be occupied by other males [29], [51]. Gavriv [52] documented arenas occupied for 55 consecutive years, corresponding to 6–18 generations in natural conditions. Our study arenas were already known by hunters in the 1970s [31]. Fidelity might result from the use of cues of conspecific occupancy as an indicator of habitat quality and mate availability, an adaptive mechanism that permits young individuals to reduce the costs of inexperience in assessing environmental quality [53], [54]. Indeed, first year Capercaillie males visit display arenas but usually do not perform full display behavior: they occupy peripheral grounds until a dominant male abandons its arena [55]. In the long run, this mechanism leads to the persistence of traditional arenas, limits the colonization of novel grounds, and contributes to maintain arenas in the oldest forest stands. The latter are also richer in bird species than younger stands, thus explaining the Capercaillie-diversity association at this scale.

### Association between male sexual displays and local bird diversity

The spatial association between individual sexual behavior and diversity values can be explained by evoking environmental processes involving gradients in habitat quality and additive effects between territorial and individual quality: The acoustic features associated with the most diverse spots have been shown to indicate large body mass (low minimum frequency) or male condition (rapid song rate) and are used by females and competitors as indices of male quality in many vertebrate species [38], [56]. Shifts in song frequencies are indirectly caused by selection for larger body size, which determines an allometric increase of the syringeal membranes and vocal tract, in turn causing the decrease of fundamental frequencies [40], a phenomenon that is most evident in dimorphic animal species [57]. Indeed, Capercaillie minimum song frequencies (280–400 Hz) border the lower limit for a bird that weighs less than 5 kg [58], and a requirement of honest traits is precisely that of laying near the individual physical limits [59]. Moreover, male neck is held upright and stretched while singing, a behavior that increases the length of the vocal apparatus and shifts song frequencies downwards. Song rate is another good candidate for a costly character, as it correlates with oxygen consumption [60]. Vigorous displaying sage grouse males expend twice as much energy per day as males that do not display [61], and females preferentially mate with lekking males calling at a faster rate [62]. In another Galliform, the grey partridge, song rate is affected by the level of testosterone [63]. In the Capercaillie, song rate increases with the number of neighboring males thus being likely used as a signal of intimidation.

### From the behavior of individuals to community properties

This study shows that the value of surrogate species depends on the spatial scale considered, and on the area of the territory that is taken into account. Many species become more intolerant passing from sites used for resting to foraging to reproduction, as these activities may impose increasingly higher costs and risks for the individual [64]. In these cases, gradients of habitat quality (or disturbance) within territories may be accompanied by oscillations in diversity values, so that the strength of the species association with the community may vary greatly, especially when the latter is composed by species with reduced spatial requirements with respect to the surrogate species (as in the case of umbrella taxa).

By considering variation at several levels of the biological hierarchy at a time, we highlighted the importance of specific phenotypic traits that may functionally explain individual-community coupling. The association between individual and territory quality likely underlies the covariation between condition-dependent sexual traits and community diversity, since high quality Capercaillies might successfully exclude competitors from long-lasting arenas, which are also those supporting the highest bird diversity. In many bird species, the best quality individuals outcompete lower quality ones and occupy the best territories, leading to correlations between honest sexual signal variation and territory quality [65]. Although in this study we illustrate an example involving a forest ecosystem, our findings and approach could be easily extended to other environments, and to a wide variety of animal species possessing costly sexual characters.

## Supporting Information

Table S1 **List of the bird species that compose the study community, ranked according to the frequency of their occurrence.**
(DOC)
