# Supplementary material for: Mechanisms Underlying the Bioindicator Notion: Spatial Association between Individual Sexual Performance and Community Diversity
Source: PLoS One. 2011 Jul 26;6(7):e22724. doi: 10.1371/journal.pone.0022724 (PMC3144237; doi:10.1371/journal.pone.0022724)
Supplement: Table S1 — List of the bird species that compose the study community, ranked according to the frequency of their occurrence. (DOC) [file pone.0022724.s001.doc]

Table S1. List of the bird species that compose the study community, ranked according to the frequency of their occurrence.

| Latin name | Frequency of  occurrence |
| --- | --- |
| *Erithacus rubecula* | 69.9 |
| *Parus ater* | 65.7 |
| *Troglodytes troglodytes* | 41.1 |
| *Fringilla coelebs* | 39.0 |
| *Sitta europaea* | 32.2 |
| *Parus major* | 23.3 |
| *Certhia brachydactyla* | 22.5 |
| *Regulus ignicapillus* | 22.0 |
| *Sylvia atricapilla* | 20.8 |
| *Parus caeruleus* | 20.3 |
| *Parus palustris* | 18.2 |
| *Regulus regulus* | 14.8 |
| *Prunella modularis* | 12.7 |
| *Parus cristatus* | 11.9 |
| *Certhia familiaris* | 8.5 |
| *Garrulus glandarius* | 7.6 |
| *Aegithalos caudatus* | 5.5 |
| *Dendrocopos major* | 5.5 |
| *Columba palumbus* | 5.1 |
| *Turdus philomelos* | 5.1 |
| *Turdus merula* | 4.7 |
| *Scolopax rusticola* | 3.4 |
| *Turdus viscivorus* | 2.1 |
| *Phylloscopus ibericus + P. collybita* | 1.7 |
| *Anthus trivialis* | 0.8 |
| *Pyrrhula pyrrhula* | 0.8 |
| *Cuculus canorus* | 0.8 |
| *Motacilla cinerea* | 0.8 |
| *Dendrocopos medius* | 0.8 |
| *Dryocopus martius* | 0.8 |
| *Phoenicurus phoenicurus* | 0.4 |
| *Emberiza cia* | 0.4 |
| *Motacilla alba* | 0.4 |
| *Sylvia hortensis* | 0.4 |
| *Serinus citrinella* | 0.4 |
